# Report on the Medicinal Use of Eleven Lamiaceae Species in Lebanon and Rationalization of Their Antimicrobial Potential by Examination of the Chemical Composition and Antimicrobial Activity of Their Essential Oils

**DOI:** 10.1155/2016/2547169

**Published:** 2016-12-08

**Authors:** Madona Khoury, Didier Stien, Véronique Eparvier, Naïm Ouaini, Marc El Beyrouthy

**Affiliations:** ^1^CNRS, Institut de Chimie des Substances Naturelles, UPR 2301, 1 Avenue de la Terrasse, 91198 Gif-sur-Yvette, France; ^2^Department of Agricultural Sciences, Holy Spirit University of Kaslik, Kaslik, B.P. 446, Jounieh, Lebanon; ^3^Sorbonne Universités, UPMC Univ Paris 06, CNRS, Laboratoire de Biodiversité et Biotechnologies Microbiennes (LBBM), Observatoire Océanologique, 66650 Banyuls-sur-mer, France

## Abstract

Many Lamiaceae species are consumed in the Lebanese cuisine as food or condiment and are largely used in the traditional medicine of Lebanon to treat various diseases, including microbial infections. In this article we report the traditional medicinal uses of eleven Lamiaceae species:* Coridothymus capitatus* L.,* Lavandula stoechas* L.,* Lavandula angustifolia* Mill.,* Mentha spicata* L. subsp.* condensata*,* Origanum syriacum* L.,* Rosmarinus officinalis*,* Salvia fruticosa* Miller.,* Satureja cuneifolia* Ten.,* Satureja thymbra* L.,* Thymbra spicata* L., and* Vitex agnus-castus* L. and study the chemical composition and antimicrobial activity of their essential oils (EOs). Our survey showed that Lamiaceae species are mainly used against gastrointestinal disorders and microbial infections. Chemical analysis of the EOs obtained from these plants allowed us to identify seventy-five compounds describing more than 90% of the relative composition of each EO. Essential oils with high amounts of thymol and carvacrol possessed the strongest antimicrobial activity. As expected, these two compounds demonstrated an interesting antifungal efficacy against the filamentous fungus* T. rubrum*. Our results confirmed that some of the Lamiaceae species used in Lebanon ethnopharmacological practices as antimicrobial agents do possess antibacterial and antifungal potential consistent with their use in alternative or complementary medicine.

## 1. Introduction

The evolution of resistance to currently used antimicrobial compounds is neither a surprising nor a new phenomenon; however, infections are becoming more common, more severe, and more easily transmitted. According to the WHO, many infectious diseases will become untreatable and uncontrollable in the upcoming years [1]. Many authors mention a possible upcoming postantibiotic era [2–4].

Aromatic plants have been recognized since antiquity and widely used as bactericides, fungicides, virucides, antiparasitics, and pesticides. Their properties are mainly attributed to their volatile oils [5, 6]. Investigations into the antimicrobial activities, mode of action, and potential uses of plant essential oils have regained momentum [7]. These oils, representative of very wide chemical diversity, may contribute to or inspire alternative solutions against multidrug resistant infections. Recently,* in vitro* screening programs based on ethnobotanical approaches proved to be very efficient in validating traditional uses of medical plants and providing new ways in the search for active compounds [8].

Lamiaceae (formerly known as Labiatae) is a large plant family of mostly shrubs and herbs. It is the largest family of the order Lamiales with 236 genera and more than 7,000 species, the largest genus being* Salvia* with around 900 species [9]. Lamiaceae are distributed globally and a particularly high concentration of them occurs in the Mediterranean region. The majority of Lamiaceae being aromatic plants, the family is economically important [10]. Many are cultivated as ornamentals, like* Ajuga, Coleus,* and* Salvia* but others are widely used as culinary herbs and spices, such as sage (*Salvia*), thyme (*Thymus*), mint (*Mentha*), oregano or marjoram (*Origanum*), rosemary (*Rosmarinus*), lavender (*Lavandula*), and basil (*Ocimum*). Mint and lavender are grown for their oil used in perfumery, cosmetics, pharmaceutical, and food industries as active ingredients or as flavour and fragrance. Medicinal properties of the Lamiaceae species are often attributed to their high content of volatile compounds.

The Lamiaceae family is particularly well represented in Lebanon, where 136 species belonging to 29 genera have been inventoried [11]. Many Lamiaceae are regularly consumed in the Lebanese cuisine as food or condiments. For example, the different varieties of thyme like* Origanum syriacum*,* Satureja thymbra,* and* Thymbra spicata* associated with a mixture of* Rhus coriaria* L. (sumac) and sesame seeds are the main ingredients of a very popular Lebanese pizza called “manakeesh.” Others, like* Rosmarinus officinalis*,* Coridothymus capitatus*, and* Salvia fruticosa,* are eaten as salads and the leaves of* Thymus* and* Origanum* species are mixed with traditional Lebanese fresh cheese called “Shanklish” for their aromatic and antiparasitic properties.

Indigenous Lamiaceae are also frequently used in Lebanon for medical purposes and are marketed by herbalists. These include the genera* Lavandula*,* Melissa*,* Mentha*,* Origanum*,* Rosmarinus*,* Salvia*,* Satureja,* and* Thymus*. These plants are highly aromatic due to the presence of external glandular structures that produce volatile oil [12] and their essential oils are widely used in the Lebanese folk medicine [11, 13, 14].

However, there was no previous study documenting the folk medicinal usage of the Lamiaceae species in Lebanon. We have conducted a large-scale survey on the traditional medicinal uses of Lamiaceae species in different regions of Lebanon. A part of this work has already appeared in a congress report [11].

The main objectives of the present study were (1) to report on the traditional uses of eleven Lamiaceae species most used in the Lebanese folk medicine (*Coridothymus capitatus* L. Reichenb. Fil.,* Lavandula stoechas* L.,* Lavandula angustifolia Mill.*,* Mentha spicata* L. subsp.* condensata*,* Origanum syriacum* L.,* Rosmarinus officinalis* L.,* Salvia fruticosa* Miller.,* Satureja cuneifolia* Ten.,* Satureja thymbra* L.,* Thymbra spicata* L., and* Vitex agnus-castus* L.), (2) to investigate the chemical composition of the EOs extracted from these species, (3) to evaluate the antimicrobial activity of these EOs and their major constituents against opportunistic human pathogens, and (4) to correlate* in vitro* results with the ethnopharmacological uses of these plants.

## 2. Material and Methods

### 2.1. Ethnomedical Field Survey and Ethnobotanical Data Collection

**Figure 1:**
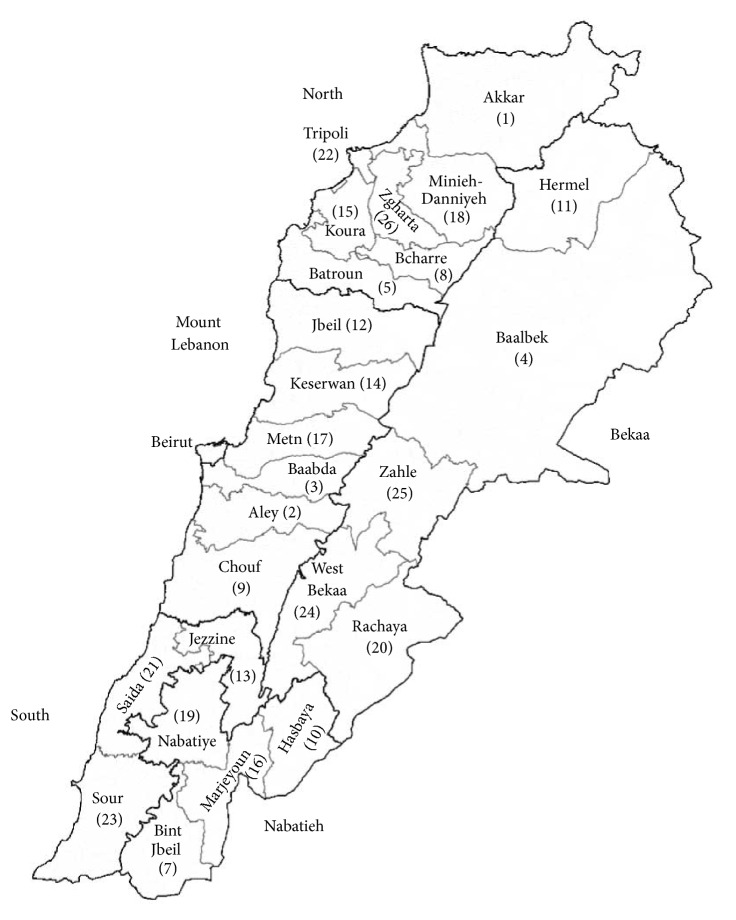
Studied area: the governorates and districts of Lebanon.

The research was carried out in different regions of Lebanon from the north to the south and from the coast to the mountains, including the anti-Lebanon mountain range and the steppe. A great variety of wild plant species according to different ecological conditions can therefore be found. The surveys were conducted in the cities and villages of the twenty-five districts (“aqdya” or “qadaa”) of the six governorates (“mohaafazah”) of Lebanon (Figure 1).

Ethnobotanical and ethnomedicinal information was obtained between the years 2002 and 2008 from 325 interviewees from 223 villages covering all the districts of Lebanon. Most of the participants interviewed were herbalists (“Attarin” or “dabbous”), shepherds, farmers, folk healers, or older experienced people and midwives (“daye”) between 40 and 70 years old. The people interviewed declared that their knowledge about the traditional medicine was transmitted mainly orally from older generations. Interviewees were accompanied to the field individually where they would point out the herbs that have been using to cure the mentioned disease. This was also to confirm plant identification (vernacular names can be different in different regions of Lebanon) and obtain fresh samples for herbarium voucher. When the fertile part was not available, the plant was visited again at the appropriate time to obtain a fertile sample.

The obtained information was cross-checked with that of other informants, and also, after a week, the interview was repeated with the same person. Only medical usages of plants given at least by three separate informants have been reported in this investigation. It was verified that each informant is able to recognize the mentioned plant species in the wild.

The questionnaire form was based on the botanical and ethnopharmacognosic survey of traditional medicine plants, suggested by WHO [15]. The collected information included local names, used part(s) of the plant, folk medicinal uses and therapeutic properties, method of preparation, way of administration, doses, and duration of treatment. The results are recorded in a synoptic table (Table 2).

### 2.2. Plant Material

Plant material was collected in several locations throughout Lebanon and voucher specimens were deposited at the Herbarium of Botany of the U.S.E.K., Lebanon. Specimens of the Lamiaceae species were collected as described in Table 1.

The plants have been identified based on the “Nouvelle flore du Liban et de la Syrie” (Mouterde) [16] and the Med-Checklist (http://ww2.bgbm.org/mcl/). We followed the new phylogenetic classification APG II [17] in order to update the families cited in Mouterde. Voucher specimens were deposited at the Herbarium of Botany of the U.S.E.K., Lebanon.

### 2.3. Essential Oil Extraction

The essential oils (EOs) were obtained by hydrodistillation performed for 3 h using a Clevenger-type apparatus according to the European Pharmacopoeia [18]. Yields are given in Table 4.

### 2.4. Essential Oils Analyses

#### 2.4.1. GC Analyses

Analytical gas chromatography was carried out on a Thermo Electron Corporation gas chromatograph fitted with a DB-5 MS capillary column (30 m × 0.25 mm) with 0.1 *µ*m film thickness or a fused silica HP Innowax polyethylene glycol capillary column (50 m × 0.20 mm, film thickness 0.20 *µ*m). Helium was the carrier gas (0.7 mL/min). The column temperature was initially set to 35°C before being gradually increased to 85°C at 5°C/min, held for 20 min at 85°C, raised to 300°C at 10°C/min, and finally held for 5 min at 300°C. Diluted 1 *µ*L samples (1/100, v/v) were injected at 250°C manually and in the splitless mode. Flame ionisation detection (FID) was performed at 310°C.

#### 2.4.2. GC/MS Analyses

The GC/MS analyses were performed using an Agilent 6890 gas chromatograph coupled with 5975 Mass Detector. The 7683B autosampler injected 1 *µ*L of each oil sample. A fused silica capillary column DB-5 MS (30 m × 0.25 mm internal diameter, film thickener 0.1 *µ*m) or a fused silica HP Innowax polyethylene glycol capillary column (50 m × 0.20 mm, film thickness 0.20 *µ*m) was used. Helium was the carrier gas (0.7 mL/min). The oven temperature program was identical to that described in Section 2.4.1. The mass spectra were recorded at 70 eV with an ion source temperature of 310°C and a transfer line heated to 320°C. The acquisition was recorded in full scan mode (50–400 amu).

#### 2.4.3. Identifications and Quantifications

Most constituents were identified by gas chromatography by comparing their retention indices (RI) with those from the literature [19, 20] or with those of authentic compounds obtained from Sigma-Aldrich (Lebanon). The retention indices were determined relatively to a homologous series of* n*-alkanes (C8 to C24) analysed under the same operating conditions. Further identification was obtained by comparing their mass spectra on both columns with those provided in the NIST and Wiley 275 libraries, our homemade library constructed with pure compounds, and EOs of known composition or with mass spectra from the literature [19, 21]. The relative concentrations of the components were calculated based on the GC peak areas without correction; they are reported in Table 4.

### 2.5. Antimicrobial Activity

#### 2.5.1. Microorganisms

The antimicrobial activity of the essential oils was investigated against the Gram (−) bacterial strain* Escherichia coli* ATCC 25922, the Gram (+) bacterial strain* Staphylococcus aureus* ATCC 29213, the yeast* Candida albicans* ATCC 10231, and a clinical isolate of the dermatophyte* Trichophyton rubrum* SNB-TR [22].

#### 2.5.2. Microdilution Method

The antimicrobial activity of the EOs was measured using a broth microdilution method according to the Clinical and Laboratory Standards Institute (CLSI) guidelines [23–26]. The essential oils and their major components were diluted in DMSO and were tested at concentrations ranging from 512 to 16 *μ*g/mL. The microplates were incubated at 37°C for 24 h for bacteria, 48 h for yeasts, and 5 days for dermatophytes. The minimal inhibitory concentrations (MIC) refer to the lowest concentrations preventing visible microbial growth (Table 5). Oxacillin and gentamicin (16—0.03 *μ*g/mL) were used as reference antibiotics, while itraconazole (16—0.03 *μ*g/mL) and fluconazole (64—0.125 *μ*g/mL) were used as positive controls for the antifungal assays. The antimicrobial standards were purchased from Molekula, Dorset, UK, and the pure terpenes from Sigma-Aldrich, Lebanon.

## 3. Results and Discussion

### 3.1. Ethnopharmacological Data

This investigation showed that Lamiaceae species and especially the eleven ones reported in this study are still frequently used in Lebanon as herbal remedies (Table 2).* Origanum syriacum* and* Salvia fruticosa* are the two most cited plants. It should be noted that the same vernacular name is sometimes used for different species. For example, the appellation “Za'atar” is used for* Satureja thymbra*,* Thymbra spicata*,* Coridothymus capitatus*, and* Origanum syriacum*.

### 3.2. Plant Parts Used

The plant parts used most frequently are the flowering tops and the leaves (34.6% each), followed by the stems (11.5%), flowers (7.7%), whole plant, fruits, and seeds (3.8% each) (Table 3).

### 3.3. Preparation and Administration

Most of the traditional remedies are administered* per os* or internally (71.8%) although the majority of the EOs are applied externally (57.1%). The main modes of preparation are infusion (40.0%), decoction (17.5%), EO application (17.5%), and food consumption (15.0%) (Table 3). The contribution of the volatile organic compounds (VOCs) in all of these preparations is expected to be very important owing to the large amount of VOCs in Lamiaceae. The only exception is for decoctions because this process should eliminate most of the VOCs along with steam.

### 3.4. Traditional Medicinal Indications

Among the multiple medical usages of these plants, the eleven Lamiaceae species are mainly used to treat gastrointestinal disorders and microbial infections (Table 3). Almost all of them are described to possess antiseptic, antimicrobial, or antifungal properties, and a significant proportion of the EOs has been reported to be applied locally to cure microbial infections (57.1%).

### 3.5. Number of Districts Describing the Use of Each Lamiaceae Species

The ethnopharmacological use of* Rosmarinus officinalis* and* Origanum syriacum* was mentioned by the largest number of districts (57.7 and 50%, resp.), while the medicinal use of* Coridothymus capitatus*,* Vitex agnus-castus,* and* Salvia fruticosa* was localized in certain regions of Lebanon (Table 3).

### 3.6. Comparison with the Ethnopharmacological Uses in Other Mediterranean Countries

Despite the ancestral use of medicinal plants including essential oils in Lebanon, literature reports on Lebanese ethnomedicinal practices are scarce. Nevertheless, previous works have reported the medicinal uses of Lamiaceae species in the Mediterranean basin. Indeed, some of the plant species pointed out in our study are widely distributed and widely acknowledged by local people for medicinal purposes. In many cases, the uses reported in other countries are somewhat comparable to those found in Lebanon. For example, the cultivated lavender* Lavandula angustifolia* is used in Italy and Turkey as a sedative and antiseptic and to treat cold and rheumatism [27–29]. As for the wild lavender,* Lavandula stoechas*, it is used in Turkey and Spain for gastrointestinal and cardiovascular diseases [30–34]. Other examples include* Coridothymus capitatus* and* Salvia fruticosa* used in Palestine and Israel to treat cold and gastrointestinal disorders [35–37],* thymbra spicata* used in Turkey for its cardiotonic and hypoglycemic properties, or to treat cough and arthrosclerosis [29, 30, 32],* Vitex agnus-castus* used in Palestine to treat eye inflammation [38], and* Mentha spicata* and* Rosmarinus officinalis* that are both used in Italy in the same way as in Lebanon as antiseptics and antimicrobials [27, 39].

On the other hand, little is known on the plant species with restricted distribution areas, like* Origanum syriacum* that is mainly used to treat cold symptoms in Turkey [40] and stomach pain in Jordan [41]. It is also the case of* Satureja cuneifolia* and* Satureja thymbra* that are used in Turkey as immunotonic and cardiotonic and to treat cold and flu symptoms [32, 42].

### 3.7. Essential Oils Analyses

Since the VOCs seemed to be relevant in the context of the traditional medicinal use of Lebanese Lamiaceae, it was pertinent to study the chemical composition of their EOs. These are reported in Table 4; the extraction yields and the relative proportions of the components are given.

The yields (v/w, relative to dry weight material) obtained by hydrodistillation ranged from 0.3% from* V. agnus-castus* flowering tops to 4.1% from* T. spicata* leaves. GC and GC-MS analyses led to the identification of 75 components accounting for 90.6 to 97.2% of the total oils (Table 4). It was found that the EOs were essentially composed of oxygenated monoterpenes (51.7% to 85.9%), the only exception being that from* Vitex agnus-castus* which was mainly composed of sesquiterpene hydrocarbons (36.4%). Thymol and carvacrol were present in a high relative proportion in several EOs of these Lebanese chemotypes. Thymol was the major constituent of* Origanum syriacum* (74.4%),* Satureja thymbra* (44.5%), and* Coridothymus capitatus* (29.3%) EOs, while carvacrol was highly abundant in the EOs of* Satureja cuneifolia* (69.5%),* Thymbra spicata* (64.0%), and* Coridothymus capitatus* (29.3%). All of these species were also rich in* p*-cymene and*γ*-terpinene. The oils of* Salvia fruticosa*,* Rosmarinus officinalis,* and* Vitex agnus-castus* were essentially composed of eucalyptol representing 57.3%, 21.5%, and 20.5% of the total oils, respectively. Pulegone was the main component of the EO of* Mentha spicata* accounting for 78.7% of the oil. Wild and cultivated lavender EOs were significantly different;* Lavandula stoechas* (wild lavender) was mainly composed of *α*-fenchone (26.2%) while linalool (45.8%) was the major constituent of* Lavandula angustifolia* (cultivated lavender).

### 3.8. Comparison of the Main Components of the Lebanese Lamiaceae Species with Other Countries

The EOs of* Vitex agnus-castus*,* Lavandula stoechas,* and* Salvia fruticosa* have a very similar chemical composition compared to other countries [43–48].* Lavandula angustifolia* and* Coridothymus capitatus* Lebanese EOs are different than those from other regions. According to our study,* Lavandula angustifolia* EO contains a lower relative proportion of linalyl acetate [49] and* Coridothymus capitatus* is richer in thymol [50, 51]. The chemical composition of the EO of* Rosmarinus officinalis* is similar to that reported in Greece [52] but is different from the ones reported in Turkey and Tunisia where the main component of the Lebanese EO, eucalyptol, is absent [53, 54]. Both thymol and carvacrol chemotypes have been previously reported for our thymol-rich EOs like* Satureja* thymbra [55, 56] and* Origanum syriacum* [57] or those in which carvacrol is the main constituent such as* Thymbra spicata* [55, 58] and* Satureja cuneifolia* [59, 60]. Likewise,* Mentha spicata* oil reported in our study belongs to the pulegone chemotype, while a carvone chemotype has also been described in other regions [61, 62].

### 3.9. Antimicrobial Activity

The minimum inhibitory concentrations (MICs) of the Lamiaceae essential oils are presented in Table 5. An oil was considered active if the minimal inhibitory concentration was 128 *µ*g/mL or below [63]. The clinical isolate* T. rubrum* SNB-TR1 was the most sensitive, followed by the Gram (−) bacterium* S. aureus *and the yeast* C. albicans*, whereas the Gram (+) bacterium* E. coli* was more resistant. The oil of* Coridothymus capitatus* was the only EO to possess a significant antibacterial activity against* E. coli* with a MIC value of 128 *µ*g/mL. This EO is rich in both thymol and carvacrol (29.3 and 41.5%, resp.). Overall, plants EOs with high relative amounts of thymol and/or carvacrol were the most active against* S. aureus*,* T. rubrum,* and* C. albicans* with MIC values in the range of 64–128 *µ*g/mL.

To confirm the origin of the observed antimicrobial activity, the major constituents were tested against the two microorganisms that showed the greatest sensitivity,* S. aureus* and* T. rubrum*. Indeed, among the tested terpenes, only thymol and carvacrol showed significant antibacterial activity against* S. aureus* (MIC 128 *µ*g/mL). Against* T. rubrum*, these two compounds were also very active (MIC 32 *µ*g/mL), followed by* p*-cymene (MIC 64 *µ*g/mL). The antimicrobial potential of a combination of thymol and carvacrol in equal proportions was also measured but no synergistic effect was detected. The MIC of the combination of these two compounds was identical to that of each separate compound. The other important constituents, that is, camphor, eucalyptol, linalool, *γ*-terpinene, and *α*-fenchone, did not show any significant antimicrobial activity (Table 5).

It has been previously reported that essential oils of* Origanum* and* Thymus* species contain mainly phenolic monoterpenes such as carvacrol and thymol and their activities are often attributed to these compounds [64–68].

Our data confirm that these plants or their EOs could indeed be used in local applications for treating mycoses and demonstrate that combinations of thymol and carvacrol are not needed to account for the antifungal property of an EO.

### 3.10. Correlation Ethnopharmacology: Antimicrobial Activity

Ten species out of the eleven most cited Lamiaceae are used in the Lebanese traditional medicine as antiseptic or antimicrobial agents. Some are even cited specifically as antifungals (3 out of 11). Our results corroborate the antimicrobial indications for* Coridothymus capitatus*,* Origanum syriacum*,* Lavandula stoechas*,* Satureja thymbra,* and* Thymbra spicata*, indicating that the use of these plant species is most likely linked to the antimicrobial potential of their VOCs. For example, the flowering parts of* C. capitatus* are used as antiseptic and to treat dermatosis, and our results showed that the EO of the flowering tops can be considered active on all strains tested. The flowering parts of* O. syriacum*,* S. thymbra,* and* T. spicata* are also used in Lebanon as antiseptic and antimicrobial agents and their EOs proved to be notably active against* S. aureus*,* C. albicans,* and* T. rubrum* (Table 5).

## 4. Conclusion

In conclusion, the ethnobotanical and ethnopharmacological survey of the Lamiaceae plants confirmed that their medical values are widely acknowledged among herbalists and rural communities and that this family of plants is still frequently used in the traditional medicine of Lebanon to treat many ailments, including microbial infections.

This study highlights the* in vitro* antimicrobial activity of some Lebanese Lamiaceae EOs against human pathogens. The antimicrobial potential of these EOs originates from their high content in either thymol or carvacrol. These results validate the traditional antimicrobial use of Lamiaceae and lead us to believe that the use of the most active ones of these plants (or their EOs) can be promoted for the treatment of mycoses under topic applications. It should be noted that Lamiaceae herbs have high consumption in the traditional medicine in Lebanon. This might indicate innocuousness [69] although toxicological studies on these species should be encouraged.

## Figures and Tables

**Table 1:** Plant material collection information.

Plant name	Part used	Collection region				Collection date	Voucher number
		Region	District	GPS Location	Altitude (m)		
*Coridothymus capitatus*	Flowering tops	Anfeh	Koura	34°21′38.69′′N 35°43′58.96′′E	8	August 2011	MNV191a
*Lavandula angustifolia*	Flowering tops	Qartaba	Mount Lebanon	34°05′39.18′′N 35°50′38.99′′E	1170	May 2012	MNC121
*Lavandula stoechas*	Flowering tops	Adonis	Keserwan	33°58′05.91′′N 35°36′35.20′′E	50	May 2012	MNV114
*Mentha spicata*	Flowering tops	Kfarzebian	Mount Lebanon	33°59′53.43′′N 35°51′29.69′′E	2015	July 2012	MNV194
*Satureja cuneifolia*	Leaves						MNV173
*Origanum syriacum*	Flowering tops	Alita	Keserwan	34°05′29.72′′N 35° 41′27.05′′E	620	August 2012	MNV188
*Rosmarinus officinalis*	Leaves	Shuwayfat	Mount Lebanon	34°48′32.43′′N 33°48′32.43′′E	200	October 2011	MNV154
*Salvia fruticosa*	Leaves	Nahr Ibrahim	Mount Lebanon	34°04′24.34′′N 35°39′19.03′′E	190	June 2012	MNV159
*Satureja thymbra*	Flowering tops						MNV173a
*Thymbra spicata*	Leaves						MNV191
*Vitex agnus-castus*	Flowering tops	Nahr Ibrahim	Mount Lebanon	34°03′42.70′′N 35°38′44.52′′E	7	June 2012	MNV096

**Table 2:** Traditional remedies from Lamiaceae plant species in Lebanon.

Scientific name	Common name	Vernacular names in Lebanon	Part used	Preparation	Traditional medicinal indication in Lebanon	*N*	Fr (%)
*Coridothymus capitatus* (L.) Reichenb. Fil	Conehead Thyme	Za'atar farisi Za'atar ‘assal Za'atar	Fp	I: infusion or decoction on an empty stomach, one cup in the morning	Gastrointestinal disorders (colics, gastritis, stomach aches, carminative) (5, 6, 9, 15)	16	4.9
					Antidiarrheal (6, 15)	6	1.8
					Antiemetic (5, 9)	5	1.5
					Antihypertensive (6, 9)	6	1.8
					Vermifuge (5, 6, 15)	4	1.2
					Spasmolytic (15)	5	1.5
					Acne (9)	4	1.2
					Antiseptic (6, 9, 15)	9	2.8
				I: cold infusion	Dermatosis (5, 15)	4	1.2
			Le	I: infusion, 1 teaspoon in a cup of water, drinking 3 cups per day	Cough, pneumonia, expectorant (5, 6, 15)	8	2.5
					Hypoglycemic (6, 15)	5	1.5
					Stomachic (5, 6)	6	1.8
					Febrifuge (6)	3	0.9

*Lavandula stoechas* L.	Spanish lavender	Astakhoudos Khuzama Shih	Fp	I: infusion, 1 or 2 cups per day	Antiseptic (2, 9, 12, 20, 22)	10	3.1
					Gastrointestinal disorders (9, 12, 20)	7	2.1
					Spasmolytic (12, 17, 22)	5	1.5
					Rheumatism (2, 20)	6	1.8
					Cardiotonic (9, 17, 22)	6	1.8

*Lavandula angustifoli*a Mill. (cultivated^*∗*^)	Common lavender or English lavender	Khuzama Lawanda	Fp Le St	I: infusion, 2 cups per day	Hypoglycemic (6, 12, 14, 18, 20)	10	3.1
					Antihypertensive (12, 18, 20)	9	2.8
					Hepatitis (14, 20)	7	2.1
					Antiseptic (6, 14, 18)	9	2.8
					Dermatitis (6, 18, 20)	8	2.5
					Migraine (12, 18)	3	0.9
					Flu (14, 18)	4	1.2
					Asthenia, stimulant (14, 20)	5	1.5
					Stomachic, antispasmodic, carminative (6, 12, 14, 18)	11	3.4
					Sedative (6, 12, 20)	4	1.2
					Uricemia (12)	3	0.9
					Diuretic (12, 18)	6	1.8
				E: EO massage	Healing (14, 17)	8	2.5
					Rheumatism (14, 17)	9	2.8
					Lumbago (17)	4	1.2

*Mentha spicata* L. *subsp. condensata* (Briq.) Greuter & Burdet	Wild mint	Na'ana'a barri	Le	I: infusion	Digestive disorders (12, 17, 20, 24, 25)	12	3.7
					Antimicrobial (12, 20, 25)	9	2.8
					Gastritis (17, 24, 25)	5	1.5
					Antiemetic (17, 24)	4	1.2
					Arthritis (12, 20)	4	1.2

*Origanum syriacum* L.	Biblical-hyssop, Lebanese oregano, or Syrian oregano	Zouba' Za'atar	Fp	I: decoction mixed with honey 2 cups per day; food mixtures	Antiseptic, antimicrobial (1, 8, 10, 11, 12, 13, 14, 26)	15	4.6
					Vermifuge (8, 11, 14)	8	2.5
					Pertussis (10, 12, 13)	6	1.8
					Antihypertensive (1, 10, 14, 26)	10	3.1
					Hypoglycemic (11, 12, 13)	7	2.1
					Stomachic (1, 13, 14, 26)	9	2.8
					Spasmolytic (8, 11, 12)	7	2.1
					Antidiarrheal (11, 13, 14)	8	2.5
					Rheumatism (1, 12, 26)	8	2.5
					Asthma (1, 8)	7	2.1
					Migraine (13, 14)	6	1.8
					Appetizer (8, 26)	5	1.5
					Stimulating memory (13)	6	1.8
			Le	I: infusion 20 g/L; 250–500 mL/day	Cough, expectorant (2, 9)	6	1.8
					Vermifuge (2)	5	1.5
					Constipation (9)	3	0.9
				I: infusion, drinking daily	Bronchitis (18, 22)	4	1.2
					Hypoglycemia (22)	3	0.9
				I: infusion, 1 teaspoon in a cup of water	Antihypertensive (12, 14)	6	1.8
					Gastrointestinal disorders (8, 12, 14)	8	2.5
				E: local application mixed with olive oil	Analgesic (2, 14)	6	1.8
					Rheumatism (2, 12)	6	1.8
					Antihypertensive (14)	5	1.5
				I: salads	Antiseptic (1, 2, 8)	6	1.8
					Antidote, blood purifying (1, 8)	4	1.2
			Fl	E: maceration and local application	Rheumatism (1, 8, 12, 14, 16, 22)	33	10.1
				E: EO mixed with olive oil and local application		18	5.5

*Rosmarinus officinalis* L.	Rosemary	Eklil-al-jabal Hasa-al-bān Nada-al-bahr	Fp Le St	I: decoction with distilled water once a day	Antiseptic (2, 12, 14, 25)	16	4.9
					Antifungal (12, 20, 25)	11	3.4
					Hypoglycemic (2, 14, 20)	13	4.0
					Stomachic (2, 12, 25)	12	3.7
					Carminative (20, 25)	8	2.5
					Antianemic (14)	7	2.1
					Febrifuge (14, 20)	4	1.2
					Constipation (2, 25)	6	1.8
					Splenomegaly (20)	5	1.5
					Emmenagogue (12, 20)	4	1.2
					Cholagogue (2, 14)	5	1.5
			Le	I: infusion 30 g/L of water, drinking one cup, 3 times a day until improvement	Liver diseases (13, 18)	5	1.5
					Arteriosclerosis, cardiotonic (7, 13)	6	1.8
					Antianemic (13)	3	0.9
					Asthma (7)	3	0.9
				E: macerated in alcohol and locally applied or leaves added to bath	Rheumatism (1, 22, 23)	28	8.6
				E: EO diluted in alcohol and water and local application	Antiseptic (4,19, 21)	26	8.0
				E: EO mixed with olive oil and applied locally to the affected areas; alcohol macerate decoction	Rheumatism (2, 12, 14)	31	9.5

*Salvia fruticosa* Miller.	Sage	Aiza'an Kassiin ‘Ouaissé Maryamiyyé	Fp Le	I: decoction	Stimulating memory (2, 3, 5, 12, 14)	8	2.5
					Hypoglycemic (2, 5)	18	5.5
					Rheumatism (3, 5, 12)	20	6.1
					Influenza (3, 14)	14	4.3
					Antihypertensive (5, 12, 14)	16	4.9
					Gastralgia (2, 12)	12	3.7
					Hepatitis (3, 5)	9	2.8
					Nephropathy (5, 12)	8	2.5
					Constipation (2, 14)	11	3.4
					Carminative (3, 14)	13	4.0
				I: 3 drops of EO added to one tablespoon of honey	Hypoglycemic (12, 14)	9	2.8
					Cough, expectorant (2, 14, 15)	13	4.0
					Spasmolytic (12)	5	1.5
					Antimicrobial, antiseptic (1, 13, 15)	10	3.1
					Febrifuge (13)	4	1.2
					Rheumatism (1, 13, 15)	8	2.5
			Le	I: decoction	Arthritis (13, 14, 15)	22	6.8
				I: infusion	Stomachic (2, 9, 12, 15)	9	2.8
					Aphrodisiac (12)	5	1.5
					Febrifuge (15)	4	1.2
					Hypoglycemic (2, 12)	7	2.1
					Carminative (9, 15)	6	1.8
					Hemostatic (9)	5	1.5
					Asthma (2, 15)	5	1.5
					Pharyngitis, laryngitis (2, 9)	6	1.8

*Satureja cuneifolia* Ten.	Wild savory	Eshabat-el- wasab Za'atar farisi	Fp St	I: infusion	Gastrointestinal disorders, (carminative, stomachic) (8, 25)	7	2.1
					Spasmolytic (25)	5	1.5
					Headache (26)	4	1.2

*Satureja thymbra* L.	Thyme-leaved savory	Za'atar khlat Za'atar bou khlayt Za'atar rumi Za'atar franji Za'atar-al-hamir Za'atar	Fp	I: infusion, EO, and food	Antiseptic, antimicrobial, antifungal (2, 3, 12)	13	4.0
					Cough (3, 12)	4	1.2
					Blood purifying (2)	3	0.9
					Anti-inflammatory (12, 14)	4	1.2
					Nephropathy (3)	3	0.9
			Le	I: infusion 50 g/L, 1 cup 3 times a day	Antidiarrheal (8, 9)	6	1.8
					Cough (9)	4	1.2
					Cardiotonic (8)	4	1.2
					Paralysis (8)	3	0.9

*Thymbra spicata *L.	Wild thyme	Za'atar khlat Za'atar bou khlayt Za'atar	Fp	I: infusion, EO, and food	Antimicrobial, antifungal (2, 3, 20, 26)	14	4.3
					Cough (3, 20, 26)	9	2.8
					Arteriosclerosis, cardiotonic (2, 20)	8	2.5
					Blood purifying (20, 26)	6	1.8
					Insomnia (3)	5	1.5
			Wp	E: gargling or mastication	Toothache (20)	5	1.5
				I: decoction	Emmenagogue (20)	3	0.9
					Hypoglycemic (20, 21)	4	1.2
					Sterility (17, 21)	3	0.9
					Oxytocic (21)	3	0.9
				E: decoction	Ophthalmia (20, 22)	8	2.5

*Vitex agnus-castus* L.	Chaste tree	Felfel-al-raheb Kaff Mariam	Fl Ft Se	I: infusion, 1 cup daily	Vermifuge (21)	5	1.5
					Gastralgia (17)	4	1.2
					Sedative (20)	4	1.2
					Hypoglycemic (20, 21)	3	0.9
					Anaphrodisiac (17)	3	0.9
				I: decoction	Emmenagogue (20)	3	0.9
					Hypoglycemic (20)	3	0.9
					Oxytocic (17)	4	1.2
					Sterility (17, 21)	4	1.2
				E: decoction	Ophthalmia (20, 22)	8	2.5

*Part used*: Fp = flowering parts, Le = leaves, Fl = flowers, Wp = whole plant, St = stems, Ft = fruits, Se = seeds, and Dp = dried plant. *Use*: E = external use; I = internal use.

Numbers from 1 to 26 are abbreviations of the 25 districts of Lebanon and the governorate of Beirut (Beirut province is undivided): Akkar 1, Aley 2, Baabda 3, Baalbek 4, Batroun 5, Beirut 6, Bint Jbeil 7, Bcharre 8, Chouf 9, Hasbaya 10, Hermel 11, Jbeil 12, Jezzine 13, Keserwan 14, Koura 15, Marjeyoun 16, Metn 17, Miniyeh-Danniyeh 18, Nabatiye 19, Rachaya 20, Sidon (Saida) 21, Tripoli 22, Tyre (Sour) 23, West Bekaa 24, Zahle 25, and Zgharta 26.

The symbols *N* and Fr are used to indicate the number and frequency of people mentioning the indication in a single plant from the total number of interviewees; that is, Fr = (*N*/total number of interviewees) × 100.

^*∗*^All Lamiaceae species cited and reported in this table are indigenous to Lebanon, except *L. angustifolia* that is imported and cultivated.

**Table 3:** Most cited preparation and administration mode, plant parts, used and traditional medicinal indications.

*Mode of administration*		*Nb*	*%*
Internal use		23	71.8
External use		9	28.2

*Preparation*		*Nb*	**%**
Infusion		16	40.0
Decoction		7	17.5
EO application		7	17.5
Food		6	15.0
Maceration and local application		2	5.0
Gargle		1	2.5
Mastication		1	2.5

*Plant parts used*	*Abbreviated*	*Nb*	**%**
Flowering parts	Fp	9	34.6
Leaves	Le	9	34.6
Stems	St	3	11.5
Flowers	Fl	2	7.7
Whole plant	Wp	1	3.8
Fruits	Ft	1	3.8
Seeds	Se	1	3.8

*Traditional medicinal indication*		*Nb*	**%**
Gastrointestinal disorders (gastritis, spasmolytic, stomachic…)		13	12.4
Antimicrobial (antiseptic, antifungal, dermatoses)		12	11.4
Hypoglycemic		10	9.5
Rheumatism		9	8.6
Respiratory disorders (bronchitis, cough…)		8	7.6
Antihypertensive		6	5.7
Carminative		6	5.7
Vermifuge		4	3.8
Cardiotonic		4	3.8
Febrifuge		3	2.8
Flu/influenza		3	2.8
Constipation		3	2.8
Hepatitis		3	2.8
Nephropathy		3	2.8
Blood purifying		3	2.8
Various^*∗*^		15	14.3

*Number of districts describing the use of each species*		*Nb*	**%**
*Coridothymus capitatus*		4	15.4
*Lavandula stoechas*		6	23.1
*Lavandula angustifolia*		6	23.1
*Mentha spicata*		5	19.2
*Origanum syriacum*		13	50.0
*Rosmarinus officinalis*		15	57.7
*Salvia fruticosa*		9	34.6
*Satureja cuneifolia*		3	11.5
*Satureja thymbra*		6	23.1
*Thymbra spicata*		7	26.9
*Vitex agnus-castus*		4	15.4

Nb = number of times cited in Table 1; % = percentage of each citation.

^*∗*^Indications cited less than three times are listed under various indications.

**Table 4:** Composition of the essential oils of the eleven Lamiaceae species.

				*Coridothymus capitatus*	*Lavandula angustifolia*	*Lavandula stoechas*	*Mentha spicata *subsp*. condensata*	*Origanum syriacum*	*Rosmarinus officinalis*	*Salvia fruticosa*	*Satureja cuneifolia*	*Satureja thymbra*	*Thymbra spicata*	*Vitex agnus-castus*
				Flowering tops EO	Flowering tops EO	Flowering tops EO	Flowering tops EO	Flowering tops EO	Leaves EO	Leaves EO	Leaves EO	Flowering tops EO	Leaves EO	Flowering tops EO
		EOs yields (%)		1.3	2.3	0.9	0.8	1.3	0.6	2.4	2.1	2.5	4.1	0.3
R_i_^a^	R_i_^b^	Compound ID	Identification^c^											
929	1035	*α*-Thujene	R_i_, MS, CoGC	0.5	0.2	t	t	1.0	0.1	t	0.8	1.3	1.5	0.1
938	1076	*α*-Pinene	R_i_, MS, CoGC	0.3	0.7	**2.2**	0.2	0.5	**5.4**	**2.6**	0.3	1.2	0.5	**7.1**
953	1076	Camphene	R_i_, MS, CoGC	0.2	0.5	0.6	0.1	0.1	1.7	0.9	0.1	0.4	0.1	t
973	1132	Sabinene	R_i_, MS, CoGC	—	—	0.1	—	—	0.1	—	—	—	—	—
975	1312	1-Octene-3-ol	R_i_, MS	0.3	0.3	—	—	0.5	—	0.2	0.1	0.2	0.2	—
980	1118	*β*-Pinene	R_i_, MS, CoGC	0.1	0.4	0.3	—	0.1	1.9	1.5	0.1	0.4	0.1	**4.4**
993	1174	Myrcene	R_i_, MS, CoGC	0.8	0.4	0.2	0.3	0.9	0.9	1.3	0.7	1.1	0.9	1.0
1005	1188	*α*-Phellandrene	R_i_, MS, CoGC	0.2	0.1	t	0.1	0.2	—	0.1	—	0.6	0.1	0.6
1013	1159	*δ*-3-Carene	R_i_, MS	0.1	0.3	—	—	0.1	t	—	t	—	0.1	—
1013	1188	*α*-Terpinene	R_i_, MS, CoGC	1.0	—	0.2	t	1.6	0.4	0.1	1.4	1.0	1.2	0.1
1025	1280	*p*-Cymene	R_i_, MS, CoGC	**9.0**	—	—	0.1	**6.9**	—	—	**4.1**	**21.7**	**12.2**	—
1030	1203	Limonene	R_i_, MS, CoGC	—	—	—	1.0	—	—	—	—	—	**—**	—
1034	1213	Eucalyptol	R_i_, MS, CoGC	—	**8.6**	**9.5**	**—**	—	**21.5**	**57.3**	—	—	**—**	**20.5**
1045	1269	*cis-β*-Ocimene	R_i_, MS	t	**2.3**	—	—	t	—	—	t	—	t	—
1050	1253	*trans-β*-Ocimene	R_i_, MS	t	0.3	t	t	0.1	t	t	0.1	0.1	t	0.9
1057	1255	*γ*-Terpinene	R_i_, MS, CoGC	**4.0**	0.2	0.1	t	**7.6**	0.9	0.2	**13.3**	**11.1**	**11.6**	0.2
1058	1556	*cis*-Sabinene hydrate	R_i_, MS	0.5	0.1	—	1.2	0.4	0.2	0.1	0.2	0.2	0.2	0.1
1086	1265	*α*-Terpinolene	R_i_, MS	0.3	0.4	—	0.1	0.1	0.7	0.1	t	0.1	0.1	0.1
1079	1401	*α*-Fenchone	R_i_, MS	—	—	**36.2**	—	—	—	—	—	—	**—**	—
1098	1553	Linalool	R_i_, MS	0.7	**45.8**	—	—	0.2	—	—	0.1	0.9	0.1	0.3
1105	1430	*α*-Thujone	R_i_, MS	—	—	—	—	—	—	1.0	—	—	**—**	—
1115	1449	*β*-Thujone	R_i_, MS, CoGC	—	—	—	—	—	—	0.9	—	—	**—**	—
1116	1584	*α*-Fenchol	R_i_, MS	—	—	0.7	—	—	—	—	—	—	—	—
1117	—	*trans-p*-Menth-2-en-1-ol	R_i_, MS	0.1	—	—	—	—	0.2	—	—	t	t	—
1138	1475	Menthone	R_i_, MS	—	—	—	**5.1**	—	—	—	—	—	—	—
1145	1532	Camphor	R_i_, MS, CoGC	—	**11.2**	**18.0**	—	—	**15.0**	**4.8**	—	—	—	—
1167	1719	Borneol	R_i_, MS, CoGC	0.8	**7.5**	0.1	—	0.1	**7.9**	**2.1**	t	0.3	0.1	—
1176	1611	Terpinen-4-ol	R_i_, MS	1.0	**5.8**	0.2	1.7	0.2	1.0	0.5	0.2	0.2	0.3	0.3
1182	1864	*p*-Cymen-8-ol	R_i_, MS	—	—	0.2	—	—	—	—	—	—	**—**	—
1189	1706	*α*-Terpineol	R_i_, MS	0.2	—	0.3	0.2	0.1	**3.1**	**4.2**	0.2	0.1	0.1	0.7
1193	1648	Myrtenal	R_i_, MS	—	—	0.2	—	—	—	—	—	—	**—**	—
1196	1804	Myrtenol	R_i_, MS, CoGC	—	—	0.4	—	—	—	0.1	—	—	**—**	—
1215	1772	Citronellol	R_i_, MS	—	—	—	—	—	—	—	—	—	—	t
1217	1725	Verbenone	R_i_, MS	—	—	0.3	—	—	**11.8**	—	—	—	**—**	—
1217	1845	*trans*-Carveol	R_i_, MS	—	t	—	—	—	0.1	t	—	—	—	—
1227	1698	Myrtenyl acetate	R_i_, MS	—	—	1.0	t	—	0.2	—	—	—	**—**	—
1233	1662	Pulegone	R_i_, MS, CoGC	—	—	—	**78.7**	—	—	—	—	—	**—**	—
1235	1857	Geraniol	R_i_, MS, CoGC	0.1	—	—	—	—	0.1	—	—	—	**—**	0.1
1239	1607	Thymol methyl ether	R_i_, MS	1.0	—	—	—	—	—	—	—	—	**—**	—
1245	1975	Carvacrol methyl ether	R_i_, MS	0.7	—	—	—	0.1	—	—	1.4	0.1	t	—
1259	1665	Linalyl acetate	R_i_, MS	—	**4.6**	—	—	—	—	—	—	—	**—**	—
1284	1597	Bornyl acetate	R_i_, MS, CoGC	—	0.1	0.5	0.1	—	**7.0**	0.5	—	—	**—**	t
1293	2198	Thymol	R_i_, MS, CoGC	**29.3**	—	—	—	**74.4**	—	0.4	—	**44.5**	0.4	—
1299	2239	Carvacrol	R_i_, MS, CoGC	**41.5**	—	t	—	—	0.2	0.4	**69.5**	**5.3**	**64.0**	t
1333	1709	*α-*Terpinyl acetate	R_i_, MS	—	—	—	—	—	—	**2.2**	—	—	—	1.2
1343	1748	Piperitone	R_i_, MS	—	t	—	0.1	—	0.1	t	—	—	—	—
1353	2186	Eugenol	R_i_, MS, CoGC	0.1	—	—	—	t	0.2	t	t	0.1	t	—
1377	1497	*α*-Copaene	R_i_, MS,	—	—	1.1	—	—	—	—	t	—	—	—
1415	1612	*β*-Caryophyllene	R_i_, MS, CoGC	1.8	0.6	0.2	**2.8**	1.1	**6.3**	**8.3**	—	1.2	0.5	**6.6**
1437	1628	Aromadendrene	R_i_, MS	—	—	—	—	—	—	1.3	0.1	0.1	—	—
1452	1668	*α*-Humulene	R_i_, MS	—	—	0.2	0.1	0.1	**2.6**	1.5	—	—	—	0.2
1455	1689	*β*-Farnesene	R_i_, MS	0.1	—	—	0.3	—	—	—	—	—	—	**16.1**
1463	1661	Alloaromadendrene	R_i_, MS	0.1	—	0.3	—	—	—	—	—	—	—	—
1464	1685	*γ*-Gurjunene	R_i_, MS	t	—	0.2	—	—	—	0.1	t	—	—	1.2
1477	1726	Germacrene D	R_i_, MS	—	0.4	—	0.2	t	—	—	0.4	—	—	—
1487	1679	*α*-Amorphene	R_i_, MS	—	—	—	—	—	—	0.2	—	—	—	0.2
1491	1756	Bicyclogermacrene	R_i_, MS	—	—	—	0.4	—	—	—	0.6	—	—	**11.8**
1508	1741	*β*-Bisabolene	R_i_, MS	0.2	—	—	—	0.2	0.2	—	**2.1**	—	—	—
1526	1773	*δ*-Cadinene	R_i_, MS	0.1	—	1.8	t	0.1	—	0.3	0.1	t	t	0.3
1553	2076	*α*-Copaene-8-ol	R_i_, MS	—	—	**4.7**	—	—	—	—	—	—	—	—
1565	2057	Ledol	R_i_, MS,	—	—	—	—	—	—	—	—	—	—	1.9
1577	2008	Caryophyllene oxide	R_i_, MS, CoGC	0.8	0.3	1.4	0.6	0.3	0.7	0.8	—	**2.0**	0.8	—
1591	2104	Viridiflorol	R_i_, MS,	—	—	**3.6**	—	—	—	1.1	—	—	—	0.3
1625	2088	1-epi-Cubenol	R_i_, MS	—	—	1.8	—	—	—	—	—	—	—	—
1640	2188	*τ*-Cadinol	R_i_, MS	—	0.2	—	—	0.2	—	—	—	—	—	—
1642	2209	*τ*-Muurolol	R_i_, MS	—	—	1.8	—	t	—	—	t	—	—	—
1645	2145	Torreyol	R_i_, MS	—	—	1.2	—	—	—	—	—	—	—	—
1649	2256	*α*-Cadinol	R_i_, MS	—	—	—	—	—	—	—	—	—	—	0.3
1677	2256	Cadalene	R_i_, MS	—	—	0.6	—	—	—	—	—	—	—	—
1688	2229	*α*-Bisabolol	R_i_, MS, CoGC	—	0.1	—	—	t	—	—	0.1	—	—	0.2
1689	2276	Vulgarol B	R_i_, MS	—	—	—	—	—	—	—	—	—	—	**4.7**
1948	—	Cembrene A	R_i_, MS	—	—	—	—	—	—	—	—	—	—	**2.8**
1950	2603	Phytol	R_i_, MS	—	—	—	0.1	—	0.1	—	0.1	—	—	—
1974	2408	Sclarene	R_i_, MS	—	—	—	—	—	—	—	—	—	—	**5.1**
1987	2447	(*E*)-Geranyl linalool	R_i_, MS	—	—	—	—	—	—	—	—	—	—	**3.1**

		Monoterpene hydrocarbons		17.0	5.9	3.7	3.1	19.6	12.3	6.9	21.1	39.2	28.6	14.6
		Oxygenated monoterpenes		75.8	83.9	67.6	85.9	75.6	68.4	74.6	71.5	51.7	65.2	23.1
		Sesquiterpene hydrocarbons		2.3	1	4.4	3.8	1.5	9.1	11.7	3.3	1.3	0.5	36.4
		Oxygenated sesquiterpenes		0.8	0.6	15.1	0.6	0.5	0.7	1.9	0.1	2.0	0.8	7.4
		Diterpene hydrocarbons		—	—	—	—	—	—	—	—	—	—	7.9
		Oxygenated diterpenes		—	—	—	0.1	—	0.1	—	0.1	—	—	3.1

		Total identified		95.9	91.4	90.8	93.5	97.2	90.6	95.1	96.1	94.2	95.1	92.5

Notes: Bold numbers indicate the percentages higher than 2% to show main components; t = trace, less than 0.05%.

^a^Retention index on a HP-5MS column. ^b^Retention index on an Innowax column; ^c^Ri retention index identical to bibliography; MS: identification based on comparison of mass spectra. Co-GC: retention time identical to authentic compounds.

**Table 5:** Antimicrobial activity (MIC in *µ*g/mL) of the Lamiaceae oils and there major compounds based on the microdilution method.

EOs and constituents	Microorganisms			
	*S. aureus*	*E. coli*	*C. albicans*	*T. rubrum*
	ATCC 29213	ATCC 25922	ATCC 10231	SNB-TR1
*Coridothymus capitatus*	64	128	128	64
*Lavandula angustifolia*	512	>512	512	512
*Lavandula stoechas*	128	>512	512	256
*Mentha spicata*	>512	>512	>512	512
*Origanum syriacum*	128	256	128	64
*Rosmarinus officinalis*	512	>512	512	256
*Salvia fruticosa*	>512	>512	512	256
*Satureja thymbra*	128	256	128	128
*Satureja cuneifolia*	128	256	128	128
*Thymbra spicata*	128	256	128	64
*Vitex agnus castus*	512	>512	512	512
Thymol	128	n.t.	n.t.	32
Carvacrol	128	n.t.	n.t.	32
Thymol : carvacrol (1 : 1)	128	n.t.	n.t.	32
Camphor	>512	n.t.	n.t.	256
Eucalyptol	>512	n.t.	n.t.	256
Linalool	>512	n.t.	n.t.	256
*γ*-Terpinene	>512	n.t.	n.t.	512
*p*-Cymene	>512	n.t.	n.t.	64
*α*-Fenchone	>512	n.t.	n.t.	512
Oxacillin	0.5	n.t.	n.t.	n.t.
Gentamicin	n.t.	8	n.t.	n.t.
Itraconazole	n.t.	n.t.	4	<0.03
Fluconazole	n.t.	n.t.	16	2

Notes: n.t. = not tested.
